# Menstrual cycle related depressive symptoms and their diurnal fluctuations – an ambulatory assessment study

**DOI:** 10.1186/s12905-024-03438-9

**Published:** 2024-11-18

**Authors:** Hannah Klusmann, Annette Brose, Lars Schulze, Sinha Engel, Sebastian Laufer, Elise Bücklein, Christine Knaevelsrud, Sarah Schumacher

**Affiliations:** 1 Division of Clinical Psychological Intervention, Department of Education and Psychology, Freie Universität Berlin, Schwendenerstraße 27, Berlin, 14195 Germany; 2 Division of Clinical Psychology and Psychotherapy, Department of Education and Psychology, Freie Universität Berlin, Habelschwerdter Allee 45, Berlin, 14195 Germany; 3 Clinical Psychology and Psychotherapy, Institute for Mental Health and Behavioral Medicine, HMU Health and Medical University, Olympischer Weg 1, Potsdam, 14471 Germany; 4 Department of Psychiatry and Psychotherapy, University Hospital Tübingen, Calwerstr. 14, Tübingen, 72076 Germany; 5 Department of Experimental Psychopathology, Institute for Psychology, University of Hildesheim, Hildesheim, Germany

**Keywords:** Menstrual cycle, Depressive disorders, Mood disorders, Diurnal fluctuation, Ambulatory assessment, Women’s health

## Abstract

**Background:**

Reproductive mood disorders indicate that within-person variation in depressive symptoms across the menstrual cycle can be related to ovarian hormone changes. Until now, such cycle-related symptom changes have been measured once daily, even though depression research indicates systematic diurnal changes in symptoms. Further, previous research often focused on aggregated depression scores. This study examined whether three daily assessments of depressive symptoms follow similar trajectories across the menstrual cycle and investigated within-person cyclical fluctuation of all individual symptoms and the aggregated score.

**Methods:**

77 naturally-cycling participants (35 with and 42 without depressive disorder) provided three daily ratings of depressive symptoms across one menstrual cycle to evaluate individual and summarized symptoms.

**Results:**

Reliability estimates (w) of the three diurnal measurements ranged from 0.56 to 0.78. Cyclicity showed statistically significant interindividual differences for all symptoms, and individual symptoms differed significantly from each other in their magnitude of cyclicity.

**Limitations:**

Only one menstrual cycle was assessed to reduce participant burden. Further, ovulation testing dates were based on self-reported cycle lengths, and only LH (luteinizing hormone) peaks were tested without subsequent progesterone rises.

**Conclusions:**

The results highlight the need for a symptom-specific approach to assess individual variance in cyclicity of depressive symptoms. Reliability for one daily assessment can be improved by using the afternoon value, a sum score for depressiveness, or multiple items per symptom. Furthermore, this study emphasizes, that depressive symptoms can systematically change across the menstrual cycle, and it is, therefore, important to include it in depression research. Exploring female-specific risk factors of depression will enable the development of person-tailored treatments.

**Trial registration:**

The study was preregistered at ClinicalTrials.gov (NCT04086316) with the first registration on 27/08/2019.

**Supplementary Information:**

The online version contains supplementary material available at 10.1186/s12905-024-03438-9.

## Introduction

The risk for depression in women is twice as high as in men [1]. Studies have shown that this risk discrepancy for women begins with menarche and declines after menopause, suggesting that the menstrual cycle might play a crucial role in the etiology of depression [2].

The menstrual cycle in healthy female humans lasts 28 days on average and can broadly be divided into the follicular phase (onset of menses until the end of ovulation) and the luteal phase (ovulation until subsequent menses) or, more precisely, into the mid-luteal, perimenstrual, mid-follicular and periovulatory phase [3]. Across these phases, ovarian hormone concentrations (e.g., estradiol, progesterone, etc.), regulated by the hypothalamic-pituitary-gonadal (HPG) axis, fluctuate. Hormone fluctuations have been associated with physical and psychological states in some, hormone-sensitive people [4–7]. Hence, ovarian hormone fluctuations across the menstrual cycle could plausibly result in *cycle-related symptom change* [8]. Other mechanisms, such as inflammatory processes may also play an interactive role in this relationship [9]. Most studies focus on a premenstrual exacerbation of symptoms, but a mid-cycle exacerbation of various physical and affective symptoms has also been shown to occur in a subgroup of individuals [10, 11]. Cyclicity has been especially investigated in premenstrual dysphoric disorder (PMDD), but can also occur in other mental disorders [8]. For depressive disorders, Hartlage et al. [12], showed that 58% of participants experienced premenstrual exacerbation of one or more depressive symptoms. Fakhari et al. [13] reported a mid-cycle decrease in overall depressive symptoms calculated across all participants. These available longitudinal studies investigating cyclicity of depressive symptoms measure symptoms once a day [12] or once in three cycle phases [13] across one or two menstrual cycles. Daily measures are in line with the standards of menstrual cycle research, which requires daily ratings of symptoms, ideally across multiple cycles, and thereby requires comparatively high effort from participants. However, previous studies with this intensive assessment approach did not specify the time of day for each measurement, nor did they schedule multiple assessments per day. This is understandable because multiple assessments per day increase participants’ burden even further.

In addition to menstrual cyclic fluctuations, depressive symptoms can also show diurnal fluctuations, meaning systematic changes of symptoms within a day [14–16] such as a morning- or evening-low [17]. Given such diurnal patterns of depressive symptoms, the question arises whether a single daily symptom assessment can measure systematic change across the menstrual cycle well. Accordingly, systematic variance through time-of-day effects might imply the necessity for multiple measurement occasions to reliably investigate menstrual cycle effects, or to restrict daily samplings to the same timing.

Aside from this necessity to disentangle the interplay of cycle and diurnal fluctuations, a systematic investigation of single depressive symptoms is yet missing. A focus on individual symptoms opposes to the analysis of an aggregate of depressive symptoms (e.g. subsuming loss of interest and decreased concentration, etc.) as defined by the fifth edition of the diagnostic and statistical manual of mental disorders (DSM-5, American Psychiatric Association [18]). The latter has drawn the general criticism that individual symptoms can differ in their causes and underlying biology [19]. In accordance with this view, evidence from reproductive mood disorders such as PMDD, perimenopausal or postpartum depression indicates that hormonal influences can reflect differently on specific psychological symptoms [20, 21]. For example, depressed mood [22, 23], non-fatal suicidality [24], and anger/irritability [25, 26] have shown specifically pronounced perimenstrual exacerbation in women with PMDD in contrast to, for example, concentration problems [27, 28]. Crucially in view of the present study, individual symptoms were also shown to have different patterns and expressions of cyclicity (perimentstrual or mid-cycle increases of symptoms; Kiesner et al. [10, 11]. Interpreting solely a sum-score of all depressive symptoms across the menstrual cycle might thus mask individual symptoms’ variability. Therefore, a differential, symptom-specific approach is necessary to investigate menstrual cycle effects on depression. Previous studies that inform our knowledge of menstrual cyclicity in depression reported either aggregated scores for depression, focused on individual symptoms such as sleep [29–31] or suicidality [24, 32] or investigated the dichotomous occurrence of premenstrual exacerbation [12, 13]. The next step is to investigate how the full spectrum of depressive symptoms varies across the menstrual cycle while analyzing each symptom separately.

In summary, with this study, we aim to deepen the understanding of menstrual cycle patterns of depressive symptoms, using a symptom-based approach while also considering diurnal fluctuations. Further, we investigate not only premenstrual exacerbation of symptoms but also potential mid-cycle exacerbations, as introduced by Kiesner et al. [10]. Specifically, we examined *cycle-related symptom change* of overall and single depressive symptoms (aim I). Here, we hypothesized that there are statistically significant differences between participants regarding the pattern (perimenstrual vs. mid-cycle exacerbation) and individual magnitude of *cycle-related symptom change* of depressive symptoms (hypothesis 1). Participants with and without depressive disorder were then compared regarding their *cycle-related symptom change*. Further, against the background of the frequent use of the aggregated sum-scores and the associated risk for loss of information, we investigated whether individual symptoms show different trajectories across the menstrual cycle and hypothesized, that there are significant differences in *cycle-related symptom change* between single depressive symptoms (hypothesis 2).

We further aimed to investigate *cycle-related diurnal variation*, meaning whether three daily assessments (morning, afternoon, evening) follow similar trajectories (aim II).

## Materials and methods

Our aims were tested with a daily ambulatory assessment study that investigated depressive symptoms of participants with and without depressive disorders (current major depressive episode and/or persistent depressive disorder) across one menstrual cycle. The study was preregistered at ClinicalTrials.gov (NCT04086316) with the first registration on 27/08/2019 and approved by the local ethics committee at Freie Universität Berlin (ID: 003.2019). All scripts for data preparation and analysis can be derived from the openly accessible R Script at https://osf.io/9x6uc/?view_only=75c0aefe34b941a1be992eab4e97ba23.

### Participants

Participants were recruited on social media platforms, online marketplaces, online forums specialized in depression, and through the university’s official website and outpatient clinic of Freie Universität Berlin, Germany.

Inclusion criteria were age between 18 and 45 years, female biological sex (self-report), regular (+/- 2 days) menstrual cycle, and cycle length between 26 and 30 days. Exclusion criteria were hormonal contraception or psychotropic drug intake within the last six months, late-night shifts, more than one-hour time zone shift within the last month, menopause or the menopause transition, pregnancy, breastfeeding, being less than one-year post-partum, diagnoses of bipolar disorders, substance use disorder, eating disorders or schizophrenia and medications or chronic diseases that might influence hormone regulation (for a detailed list, see Appendix A). We also excluded participants with active suicidal ideation that would interfere with participation. In this case, we informed them about treatment options and offered a follow-up phone call with a licensed psychotherapist.

### Procedure

#### Screening and clinical interview

Interested participants completed an online screening questionnaire (Unipark [33]), to assess eligibility. Firstly, a consent form was filled out. Aside from inclusion and exclusion criteria, age, biological sex, education, occupation, and health-related information, such as chronic diseases, medication, and psychological disorders were assessed. Furthermore, we assessed reproductive characteristics such as previous pregnancies, average cycle length, and the date of the previous menses onset.

After completing the inclusion criteria assessed via the screening tool, a structured clinical interview was scheduled and conducted one week before menses onset to diagnose depressive disorders and to confirm inclusion criteria. Within seven days before the start of the daily ambulatory assessment, participants completed selected modules from the Structured Clinical Interview for DSM-5 Disorders (SCID-5-CV – German version [34]) by telephone.

Data were collected between January 2020 and May 2021.

#### Daily ambulatory assessment across one menstrual cycle

Following the screening and clinical interview, an ambulatory assessment was conducted across one menstrual cycle. Data collection was carried out on a study phone (Nokia 2.1 or Nokia 3.1) using the software “mobileQ” [35]. During the study period, the corresponding application sent a prompting signal at three fixed time points (9.45 am, 2.45 pm, and 7.45 pm) every day across one menstrual cycle. Responses and response times were automatically time-stamped by the program. Participants had a 30-minute time window after the prompt to participate. Assessment started three days before the expected onset of menses and ended with the onset of the next menstrual cycle. The days before menses onset were regarded as a familiarization period and were excluded from the analyses.

#### Menstrual cycle assessment

Menstrual cycle assessment and phase determination followed the recommendations by Schmalenberger et al. [3]. Ovulation was determined through luteinizing hormone (Specifically, we examined *cycle-related symptom change* of overall and single depressive symptoms (aim I).) ovulation tests (One + step, sensitivity: 20 mlU/ml). Tests were applied consistently at 5 pm for five consecutive days around the expected ovulation (based on menses onset and cycle length). Test results were immediately communicated to the study team and pictures of the test were taken for later confirmation by trained researchers. Menstrual bleeding and menstruation pain were assessed daily, asking “Did you have your menstrual bleeding today?” and “Did you experience pain due to your menstruation today?” on a dichotomous scale (Yes/No).

### Measures

#### SCID-5-CV (selected sections)

The *Structural Clinical Interview for DSM-5 Disorders* (SCID-5-CV) is a semi-structured interview for the diagnosis of the major DSM-5 disorders (Cronbach’s α > 0.7; [34]). We used the sections concerning affective episodes (Module A), psychotic and associated symptoms (Module B), substance-related disorders (Module E), and the screening questions for eating disorders (from Module I). Symptoms were rated as present or absent by a trained interviewer and diagnoses were made according to the DSM-5 diagnostic criteria.

#### PHQ-9 (adapted for ecological momentary assessment)

The severity of depressive symptoms across the menstrual cycle was measured with a version of the Patient Health Questionnaire (PHQ-9 [36]), adapted for ambulatory assessment purposes. Thereby, the nine individual symptoms characterizing depression in the DSM-5 [18] (depressed mood, diminished interest, changed appetite, sleep disturbance, reduced movement/restlessness, low energy, feeling worthless, concentration problems, suicidal ideation), as well as a summary score could be evaluated. The answering format was changed from rating symptom frequency across the last two weeks to rating the intensity in the current moment (*How much do you feel affected by the following symptoms right now?*). Symptom occurrence and severity were answered on a 6-point Likert scale from 0 - *not at all* to 5 - *very strongly*. The fully adapted version and the German original can be retrieved from Appendix B. Because Item 3 (sleep disturbance) was only assessed in the morning, it was discarded from the analyses including diurnal variance (aim II). If suicidality ratings > 1 were reported, a clinical psychologist called the participants within one business day to assess the risk and refer them to support systems such as psychotherapists.

### Analysis plan

All analyses were conducted with R version 4.2.0 [37], using packages lavaan [38], multilevelTools [39], lmerTest [40] and ggplot2 [41] for the main analyses and figures. All scripts and packages for the analyses can be retrieved from.

https://osf.io/9x6uc/?view_only=75c0aefe34b941a1be992eab4e97ba23.

#### Cycle phase standardization

To compare menstrual cycle days and phases between participants despite varying cycle and phase lengths, we standardized cycle days (days c0-c26). These standardized cycle days reflected the same phase in the menstrual cycle for each participant. The detailed procedure for standardizing the cycle day and example cycles and their recoding can be derived from Appendix C and the openly accessible R script (https://osf.io/bfqc4/?view_only=be7992a4d6544eae8b8163383b058781). The standardization procedure was founded on recommendations by Schmalenberger et al. [3]. For days c0 – c9, we selected the first ten days of the assessment cycle to represent the menstrual and mid-follicular hormone pattern (low estrogen and progesterone).

For days c10 – c16, we selected the seven days surrounding a positive LH test to represent the periovulatory hormone pattern (strong rise and fall of oestradiol and LH). If no ovulation test result was available, we estimated the periovulatory hormone pattern by using days − 17 to -11 counting back from the onset of next menses.

For days c17 – c26, we used the last 10 days of the assessment cycle before onset of next menses, representing the mid-luteal and premenstrual hormone pattern (strong rise and fall of progesterone, slight rise and fall of estrogen).

#### Analysis to investigate cycle-related symptom change (Aim I)

The extent and pattern of *cycle-related symptom change* is heterogeneous and investigating average cyclical patterns across individuals can produce underestimations or false null findings [3, 8, 11]. To address this, we adapted a method from Kiesner et al. [11], that incorporates cosine regressions in multilevel models to describe individual differences in cyclicity. The pattern of cosine functions was used based, on the one hand, on previous analyses that demonstrated its fit to cyclical symptom change of similar symptoms [10, 11]. On the other hand, it is theoretically in line with the concept of individuals sensitivity to either hormone surges (e.g., estradiol surge before ovulation) or hormone withdrawal (e.g., progesterone withdrawal premenstrually) as recently conceptualized and reviewed by Peters et al. [42]. In our analyses, cosine regression is used to analyze non-linear time-series data, providing estimates of the amplitude (strength of *cycle-related symptom change*, described by the modulus of the regression weight) and pattern (perimenstrual or mid-cycle rise of symptoms, described by the sign before the regression weight) for each individual and symptom [11]. To address aim I, we provide descriptive statistics to summarize and compare these estimates as a marker for *cycle-related symptom change* between participants, groups, and single depressive symptoms.

**Fig. 1:**
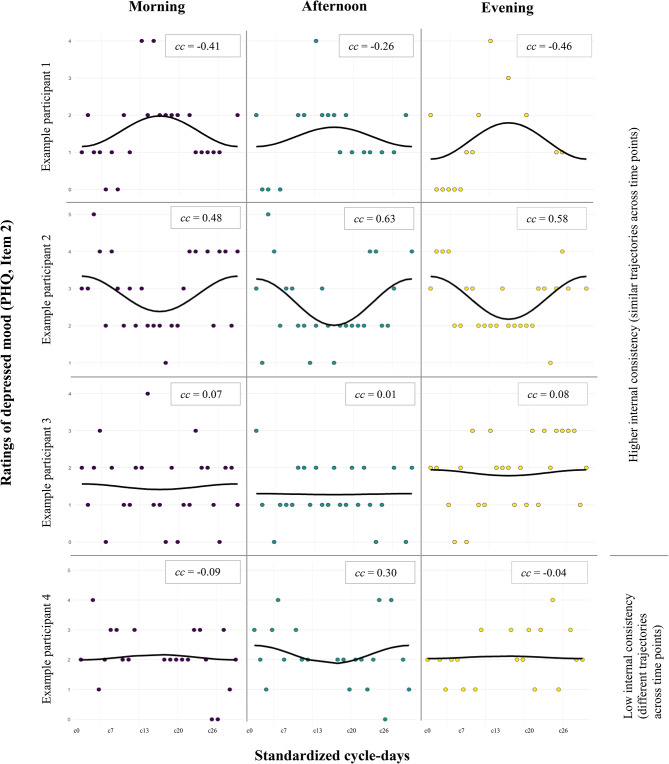
Example trajectories of depressed mood across the menstrual cycle in four participants. X-axis is scaled for standardized cycle days with c0 = onset of menses, c13 = ovulation, c26 = last day of the cycle; y-axis is absolute scale of Item 2 of the modified PHQ (symptom intensity from 0 = *not at all* to 5 = *very strongly*. cc = Cosine coefficients as measure for cyclicity for individual participant and time of day. Notes on within-person *cycle-related symptom change* (aim I): Participant 1 shows a mid-cycle exacerbation of symptoms (indicated by negative cosine coefficient), participant 2 shows a perimenstrual exacerbation of symptoms (indicated by positive cosine coefficient), participant 3 shows no exacerbation (indicated by cosine coefficient very close to zero). Notes on *cycle-related diurnal variation*/internal consistency (aim II): According to the observed scores and the smoothed lines, the symptoms of the Participant 1–3 (from top to bottom) seem to follow the same trajectories on the different daily measurement occasions. In Participant 4, instead, the trajectories do not follow the same path

In more detail, a random intercept and random effects model of the cosine function of time (time = cycle day/26 standardized cycle days) was estimated to extract the random effect of the cosine function (for examples of cosine functions see Fig. 1): $$\:{Y}_{i,j}=\:{\beta\:}_{0,j}+{\beta\:}_{1.j}\cdot\:\:\text{C}\text{o}\text{s}\text{i}\text{n}\text{e}\left[2{\pi\:}\cdot\:{\text{t}\text{i}\text{m}\text{e}}_{i,j}\right]+\:{e}_{i,j}\:$$$$\text{,}$$ with i = standardized cycle day and j = participant. If the random effect was significantly different from 0 (statistical significance calculated through 95% confidence interval of the variance), it indicated that there were statistically significant between-person differences in *cycle-related symptom change* measured by the cosine function (hypothesis 1). For all symptoms that showed such significant between-person differences, the cosine amplitude was extracted for each participant. These cosine coefficients were used as a marker for individual *cycle-related symptom change*. A positive cosine coefficient reflects a U-shaped cosine regression characterizing a perimenstrual symptom increase and a mid-cycle symptom decrease. A negative cosine coefficient reflects an ∩-shaped (inverted U) cosine regression characterizing a mid-cycle symptom increase and perimenstrual symptom decrease. The higher the modulus of the cosine coefficient, the larger is cyclicity effect in that participant (see Fig. 1 for examples).

The absolute cosine coefficients were compared between participants with and without depressive disorder using a t-test. They were further analyzed descriptively, and their distributions were compared visually with a raincloud plot - a combination of box-plot, violin plot, and jitter to visualize the distribution of all cosine coefficients of the sample.

Further, we calculated differences in cosine coefficients between symptoms to examine whether the intensity of their *cycle-related symptom change* differs (hypothesis 2). For this calculation, we applied pairwise t-tests (Bonferroni corrected for multiple testing) comparing the absolute value of cosine coefficients (the amplitudes’ modulus) of each person for each symptom.

#### Analysis to investigate cycle-related diurnal variation (Aim II)

To examine whether within-day assessments of depressive symptoms follow similar trajectories across the cycle, irrespective of the sampling timings, we compared the cyclical fluctuations of the three daily measurements and examined the assessments’ internal consistency (aim II). A high internal consistency would translate to similarly fluctuating symptoms of the morning, afternoon, and evening assessments across the menstrual cycle. A low internal consistency would translate to differently changing symptom trajectories across the cycle. Figure 1 illustrates the underlying concept of this analysis.

As a measure of within-person internal consistency, we computed the reliability index w, using multilevel confirmatory factor analyses [43]. In our analysis, the three daily assessments, measured repeatedly across the cycle, were nested within individuals. Higher reliability (w-within) indicates that the three daily assessments fluctuate more similarly across the cycle days. We calculated the reliability for each symptom and the sum score of the PHQ items^1^. Reliability estimates range between 0 and 1 and can be interpreted as no reliability (0.0–0.1), slight (0.11–0.4), fair (0.41–0.6), moderate (0.61–0.8) and substantial (0.81–1.0) reliability.

## Results

### Sample characteristics

The flowchart including the number of screened participants and reasons for exclusion can be found in Appendix D. Eighty participants met the inclusion criteria and started the study. One participant dropped out during data collection. Two participants were excluded because their menstrual cycle did not start within seven weeks after their previous menses. The remaining *n* = 77 participants were included in the statistical analyses. For demographic details, see Table 1.

Thirty-five participants met the diagnostic criteria for a current depressive disorder as assessed with the SCID-5 CV [34]. Of those, *n* = 29 met the criteria for a current major depressive episode, *n* = 12 for a persistent depressive disorder, and of those, *n* = 6 fulfilled both criteria (“double depression”).

Compliance of participants was high. On average, participants responded to 89.4% of prompts in the investigated cycle (min: 67.7%; max: 100%). After selecting standardized cycle days as described in Sect. 2.4.1, 85.8% of all theoretically possible data points and items were available for the analyses.

**Table 1:** Demographic characteristics

	Non-depressed (*N* = 42)	Depressed (*N* = 35)		Total (*N* = 77)
Age				
Mean (SD)	27.6 (5.68)	29.4 (6.41)		28.4 (6.05)
Median [Min, Max]	27.4 [18.1, 44.1]	29.1 [19.3, 42.9]		27.5 [18.1, 44.1]
**Age groups**				
18–24 years	14 (33.3%)	12 (34.3%)		26 (33.8%)
25–29 years	12 (28.6%)	7 (20%)		19 (24.7%)
30–34 years	14 (33.3%)	8 (22.9%)		22 (28.6%)
35–39 years	0 (0%)	6 (17.1%)		6 (7.8%)
40–45 years	2 (4.8%)	2 (5.7%)		4 (5.2%)
**Children**				
No children	28 (90.5%)	31 (88.6%)		69 (89.6%)
Children	4 (9.5%)	4 (11.4%)		8 (10.4%)
**Relationship status**				
In a relationship	21 (50.0%)	18 (51.4%)		39 (50.6%)
Married	2 (4.8%)	3 (8.6%)		5 (6.5%)
Single	19 (45.2%)	13 (37.1%)		32 (41.6%)
Other	0 (0%)	1 (2.9%)		1 (1.3%)
**Education**				
College/University Degree	21 (50.0%)	14 (40.0%)		35 (45.5%)
Vocational Training	0 (0%)	2 (5.7%)		2 (2.6%)
High school	20 (47.6%)	17 (48.6%)		37 (48.1%)
Middle school	0 (0%)	2 (5.7%)		2 (2.6%)
**Job**				
Other	9 (21.4%)	17 (48.6%)		26 (33.8%)
Student	33 (78.6%)	18 (51.4%)		51 (66.2%)
**Cycle length (of observed cycle)**				
Mean (*SD*)	28.0 (3.09)	28.4 (2.95)		28.2 (3.01)
Median [Min, Max]	28.0 [24.0, 43.0]	28.0 [23.0, 38.0]		28.0 [23.0, 43.0]
**Ovulation detected with LH test**				
No	19 (45.2%)	12 (34.3%)		31 (40.3%)
Yes	23 (54.8%)	23 (65.7%)		46 (59.7%)
**Depressive symptoms (Mean + SD)**				
PHQ-9 score^a^	6.21 (3.94)	15.3 (4.07)		10.3 (6.10)
SCID score^b^	1 (1.41)	5.54 (1.65)		3.08 (2.72)

Note: ^a^PHQ-9 sum-score at screening, statistically significantly higher in depressed participants compared to non-depressed participants (*t*(76) = -10.34, *p* < .001). ^b^Reported number of symptoms of major depressive episode in the SCID-5 CV interview (out of 9)

### Investigation of *cycle-related symptom change* of depressive symptoms (aim I)

To address aim I, we analyzed *cycle-related symptom change* of depressive symptoms and compared them between participants and symptoms.

*Cycle-related symptom change* was found to have statistically significant between-person differences in all assessed depressive symptoms when modelled by the cosine function including random effects. This indicates that averaging a cosine function across all participants does not adequately describe individual change; instead considering between-person differences in *cycle-related symptom change* is necessary. The parameter estimates from the multilevel models can be derived from Table 2. Because all symptoms showed significant between-person differences in *cycle-related symptom change*, all symptoms were further analyzed and their patterns were described on an individual level.

The comparison of cosine coefficients yielded no statistically significant difference between the depressed and non-depressed groups, except for symptoms of diminished interest (*p* = .042, d = 0.47) with higher mean absolute cosine coefficients in the depressed group (mean = 0.15 ± 0.07) compared to the non-depressed group (mean = 0.11 ± 0.08), and suicidality (*p* = .409, d = -0.65) with slightly higher mean cosine coefficients in the non-depressed group (mean = 0.02 ± 0.03) than in the depressed group (mean = 0.01 ± 0.01). Results including effect sizes for all symptoms can be derived from Appendix F.

The two cyclical patterns (perimenstrual or mid-cycle exacerbation of symptoms) proposed by Kiesner et al. [11] could also be identified in this sample’s participants. Figure 1 provides such exemplary cycle trajectories. The majority of participants showed a positive cosine coefficient, indicating a perimenstrual worsening of symptoms (sum score: 85.7%, diminished interest: 92.21%; depressed mood: 77.92%; changed appetite: 75.32%; feeling worthless: 79.22%, concentration problems: 68.83%; reduced movement/restlessness: 81.82%; suicidal ideation: 94.81%; sleep problems: 59.74%). Across all symptoms, between 0% (suicidality) and 31.2% (depressed mood) of participants showed a cosine coefficient larger than 0.25.

**Table 2:** Parameter estimates from multilevel models on *cycle-related symptom change* across participants

Symptom	Fixed effect cosine function	CI fixed effects	SD random effect cosine function	CI for SD of random effect
PHQ-1: Diminished interest	0.13	[0.06–0.19]*	0.15	[0.02–0.23]*
PHQ-2: Depressed mood	0.16	[0.07–0.24]*	0.29	[0.21–0.37]*
PHQ-3: Sleep problems	0.05	[-0.08–0.17]	0.33	[0.21–0.46]*
PHQ-4: Low energy	0.19	[0.10–0.27]*	0.22	[0.10–0.32]*
PHQ-5: Changed appetite	0.10	[0.00–0.20]*	0.33	[0.24–0.42]*
PHQ-6: Feeling worthless	0.07	[0.01–0.14]*	0.18	[0.11–0.25]*
PHQ-7: Concentration problems	0.07	[-0.01–0.14]	0.23	[0.16–0.31]*
PHQ-8: Reduced movement/ Restlessness	0.07	[0.01–0.12]*	0.19	[0.14–0.24]*
PHQ-9: Suicidal ideation	0.01	[-0.00–0.03]	0.04	[0.01–0.06]*
Sum score	0.84	[0.42–1.21]*	1.24	[0.85–1.64]*

*Note*: If the random effect values are significantly different from 0 (statistical significance calculated through 95% confidence interval of the variance), it indicated that there were statistically significant between-person differences in *cycle-related symptom change* measured by the cosine function. CI = 95% confidence interval, SD = Standard deviation, *confidence intervals, that do not include zero are marked as significant

**Fig. 2:**
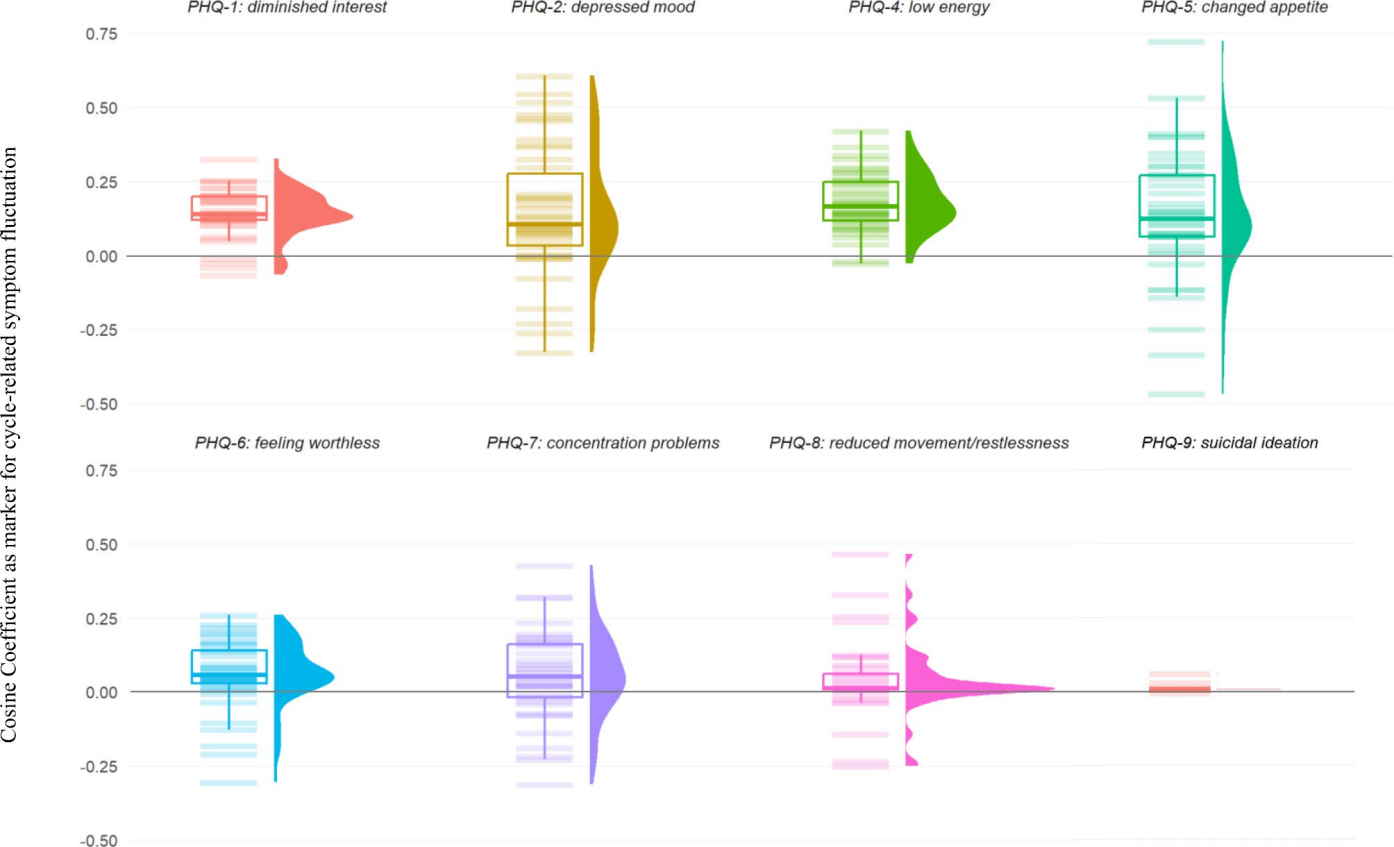
Raincloud plot of individual cosine coefficients as a marker for cycle-related symptom change of different symptoms of depression. *Note*. Mean and distribution of participants’ cosine coefficients, displayed separately for all symptoms. A cosine coefficient of zero indicates no cycle-related symptom change, a cosine coefficient above zero indicates a perimenstrual symptom worsening and a cosine coefficient below zero indicates a periovulatory symptom worsening. Larger cosine coefficients indicate stronger cycle-related symptom change. Coefficients are derived from the afternoon measurement. PHQ = Patient Health Questionnaire

Figure 2 shows a raincloud plot representing the mean and distribution of participants’ cosine coefficients, separately for all symptoms. The absolute values of cosine coefficients (as measures for *cycle-related symptom change* strength and not pattern) of individual symptoms significantly differed from each other in 19 out of 28 possible comparisons. Unsurprisingly, comparisons of suicidality (which showed low variance and low expression of cosine coefficients) yielded especially high effect sizes with strong statistical significance when compared to symptoms with rather high mean cosine coefficients such as diminished interest (see Fig. 2). The detailed results of pairwise t-tests and a visualization of their effect sizes can be derived from Appendix E

### Investigation of *cycle-related diurnal variation*: reliability of daily assessments (aim II)

The reliability estimates indicating whether depressive symptoms follow similar trajectories across the cycle when measured on three different daily occasions can be derived from Table 3. The sum score of PHQ items showed the highest reliability (ω = 0.78 [0.76–0.80]), indicating that its trajectories of the three diurnal assessments fluctuated relatively similarly across the menstrual cycle. At the item level, PHQ Items 2 (depressed mood), 5 (changed appetite), 6 (feeling worthless), 7 (concentration problems), and 9 (suicidal ideation) showed reliabilities between ω = 0.61 and ω = 0.70, which falls within the range previously discussed as moderate [44, 45]. Items 1 (diminished interest), 4 (low energy), and 8 (reduced movement/restlessness) showed fair reliabilities (ω = 0.56–0.60). Figure 1 shows exemplary trajectories of depressed mood (Item 2) across the menstrual cycle for all three daily measurements in four participants.

The multilevel factor analyses used for deriving reliability estimates also provide level-specific factor loadings (i.e., loadings of the three daily ratings on the time-varying latent variable [e.g., depressive mood across the cycle] and loadings of the three daily ratings on the person-level latent variable [e.g., depressive mood varying across persons]). The afternoon assessments showed the highest within-person factor loadings for all items except for Item 8 (reduced movement/restlessness). Therefore, the afternoon assessments best represent the cyclical fluctuation of depressive symptoms and were used in all following analyses.

**Table 3:** Reliability and factor loadings of within-day assessments (based on ω)

Symptom	Overall estimate ω	Morning	Afternoon	Evening
PHQ-1: Diminished interest	0.60 [0.57–0.64]	0.51	0.76	0.46
PHQ-2: Depressed mood	0.69 [0.66–0.72]	0.57	0.79	0.59
PHQ-4: Low energy	0.60 [0.57–0.64]	0.42	0.81	0.49
PHQ-5: Changed appetite	0.70 [0.67–0.73]	0.64	0.79	0.54
PHQ-6: Feeling worthless	0.69 [0.66–0.72]	0.54	0.80	0.60
PHQ-7: Concentration problems	0.65 [0.62–0.68]	0.61	0.67	0.57
PHQ-8: Reduced movement/ Restlessness	0.56 [0.52–0.61]	0.58	0.55	0.53
PHQ-9: Suicidal ideation	0.61 [0.58–0.65]	0.55	0.68	0.54
Sum score	0.78 [0.76–0.80]	0.68	0.86	0.67

*Note*: ω = reliability estimates indicating whether depressive symptoms follow similar trajectories across the cycle when measured on three different daily occasions. Higher ω indicates higher internal consistency. Reliability estimates range between 0 and 1 and can be interpreted as no reliability (0.0–0.1), slight (0.11–0.4), fair (0.41–0.6), moderate (0.61–0.8) and substantial (0.81–1.0) reliability [44, 45]

## Discussion

### Summary and interpretation of results

This study investigated menstrual cyclicity of depressive symptoms in a sample of 77 participants with and without current depressive disorder. The results generate knowledge on menstrual cycle-related biological underpinnings regarding the etiology and development of depression and other disorders that incorporate depressive symptoms.

Using an ambulatory assessment design with three measurements per day, we investigated the internal consistencies of depressive symptom trajectories when measured in the morning, afternoon and evening, across one menstrual cycle. The reliability analyses of the three diurnal assessments revealed reliability estimates ranging from 0.56 to 0.78. Except for Item 8 (reduced movement/restlessness), the three daily assessments seem to follow rather similar trajectories across the cycle.

When investigating how depressive symptoms fluctuate across the menstrual cycle (i.e., examining cyclic patterns), multi-level-models revealed statistically significant individual differences in cyclicity, confirming hypothesis 1. Cosine coefficients did not differ significantly between participants with and without depressive disorder, except for diminished interest and suicidality, with higher absolute cosine coefficients as a measure for cyclicity in depressed participants. Further, the intensity of cyclicity varied between single depressive symptoms, both descriptively (Fig. 2) and when statistically comparing cosine coefficients between symptoms. This confirms hypothesis 2.

#### Aim I: interpretation of results - within-person cyclicity of depressive symptoms

As revealed by multilevel models, depressive symptoms show statistically significant individual differences in cyclicity of depressive symptoms, confirming hypothesis 1. This is in line with previous research, in which individual differences in symptom sensitivity to hormone fluctuation were revealed repeatedly [7, 8]. This concept of hormone sensitivity stating that some individuals suffer from abnormal symptom sensitivity to normal hormone change has been investigated and strengthened in various reproductive mood disorders (i.e. premenstrual dysphoric disorder, perinatal depression, or perimenopausal-onset depression) for the last three decades [4, 6, 7, 46]. Even though the exact mechanisms for increased sensitivity to hormone change remain poorly understood, individual differences have been observed across disorders and studies. Our results show individual differences in depressive symptoms in participants with and without depressive disorders across the menstrual cycle and one possible explanation for this could be hormone sensitivity. The results emphasize that an overall measure of cyclicity, summarizing all participants, is highly problematic and can result in underestimations of individual cycle effects. This is especially relevant when taking different cyclicity patterns into account (perimenstrual vs. mid-cycle worsening of symptoms), which can cancel each other out if averaged [11].

Further, our results indicate differences in cyclicity patterns between single depressive symptoms, confirming hypothesis 2 This is in line with previous research showing that single (affective) symptoms had especially high hormone sensitive properties [11, 20, 47]. Furthermore, this emphasizes that summarizing depressive symptoms into one score can lead to a loss of information on cyclicity; symptoms with low cyclicity might mask cyclicity effects of other symptoms.

In summary, our results indicate that cyclicity is person-specific and item-specific. Future studies should use a symptom-specific approach and allow for between-person variance in cyclicity to reduce the risk of information loss or dampened effects due to averaging symptoms or participants.

#### Aim II: interpretation of results - reliability of daily assessments

The reliability estimates of the three diurnal measurements varied across symptoms with estimates ranging from 0.56 to 0.78. On the one hand, these estimates indicate, that the single measures were not perfectly consistent (ω = 1) and three daily measures improve the cyclicity measurement. On the other hand, the reliability estimates of the three daily measures were mostly moderate, indicating that the trajectories across the cycle represent the same underlying latent variable causing cyclicity. Considering practical constraints and participants’ burden, one could argue for one daily measurement when examining menstrual cycle-related fluctuation of symptoms across the menstrual cycle.

The aggregated PHQ score showed the highest reliability estimate, which is expected because an aggregated measure is less sensitive to measurement errors [48, 49]. Accordingly, when considering individual symptoms measured with single items, reliability was lower. An inspection of the factor loadings from these analyses revealed that the afternoon assessments had the highest loadings, indicating that these assessments are the best indicator of the latent variable driving symptom variability across the cycle.

The practical implications of these reliability analyses are diverse and depend on future studies’ constraints. Ideally, studies interested in day-to-day variations of individual symptoms across the menstrual cycle would measure symptoms multiple times a day. Subsequent analyses could then work with the resulting latent variable that corrects for unreliability due to within-day variation. One could even improve such study designs by including multiple items per assessment occasion. Considering participant burden and potential other constraints, such ideal designs might not be realistic. Choosing the best indicator of the latent variable, the afternoon assessment might be the next best alternative. In case practical reasons speak against planning assessments on afternoons due to more variable daily routines at this time of the day, it seems especially relevant to increase reliability through the inclusion of multiple items per symptom on morning- or evening assessments. In contrast to the PHQ-9, the Daily Rating of Severity of Problem (DRSP; Endicott et al., [50]) assesses some symptoms with multiple items (e.g. increased appetite/food cravings), and might thus be considered as an alternative for these symptoms. Future cycle research could benefit greatly from the development of even more precise item batteries that assess single depressive symptoms.

In summary, these results show that if one is interested in investigating cycle-related variation of depressive symptoms, multiple items for each symptom and/or multiple daily assessments can be recommended to gain more precise results. At the same time, reducing assessments and the number of items is especially relevant in menstrual cycle research where daily measures are necessary, ideally across multiple cycles, and the burden for participants is high. Using one measurement timepoint – either with an aggregated measure, measuring in the afternoon, and/or using multiple items per symptom - can optimize feasibility and should yield reliable measurements.

### Limitations

The first limitation of the study is that only one menstrual cycle was investigated per subject. Measuring at least two cycles would have been preferable, but the burden of multiple daily questionnaires for one cycle was already high. The implications of this paper contribute to the future possibility of using one daily measure while still achieving reliable results (e.g., through focusing on the afternoon assessments while working with sum scores or multiple items per symptom).

Secondly, the group comparison is limited by a solely dichotomous group assignment. Even though the SCID-CV is the gold standard in assessing depressive disorders [51], a number of participants reported subclinical depressive symptoms. Therefore, a more dimensional approach would be preferred in future studies with larger power to determine the precise associations between depressiveness and cyclicity.

Thirdly, limitations need to be considered regarding two items from the PHQ-9: reduced movement/restlessness (Item 8) and suicidality (Item 9). Participants reported confusion about Item 8 because it incorporates two experiences in one – reduced movement *or* restlessness. This resulting ambiguity might have caused the rather low reliability estimate (ω = 0.56). Therefore, the results of reduced movement/restlessness need to be considered with caution. Further, the item on suicidality showed very low variance across assessments (e.g. see Fig. 2). This is most likely caused by active suicidal ideation being an exclusion criterion for this study. Therefore, it is not possible to provide generalizable statements on the cyclicity of suicidal ideation based on this sample. However, as reviewed by Owens and Eisenlohr-Moul [32], there are indications for cyclical fluctuations of suicide risk and this should be further considered in future studies. Additionally, practical and ethical reasons to monitor suicidality in a clinical study on depressive symptoms might speak for including the suicidality item regardless of its poor reliability and low variance.

Lastly, the time frame for ovulation testing was based on self-reported cycle length and regularity. While self-reported cycle length has regularly been shown to be sufficiently reliable, previous research revealed higher irregularity in samples with depressive disorders [52]. Accordingly, it is possible that the time windows for ovulation testing were not accurately classified due to a higher chance of irregular cycles. Furthermore, to confirm ovulation, only LH rise was assessed and no additional ovarian steroid concentrations were measured. While this procedure complies with current guidelines on menstrual cycle assessment, daily measures of ovarian steroid concentrations should be added in future studies to confirm ovulation through a rise in progesterone.

### Conclusion

On a larger scale, this study emphasized, that depressive symptoms can be systematically influenced by the menstrual cycle and therefore it is of utmost importance to include the menstrual cycle in research on depressive symptoms. For example, Exploring PME trajectories and PHQ-cut-offs in such a sample is a very important next step. It is highly imperative to increase our understanding of female-specific factors of depression, as this may reduce personal burden, and enhance both efficacy and cost-efficiency of mental health care for women. Research on the role of ovarian steroids and the menstrual cycle in depression etiology leaves many open questions, such as the exact biological underpinnings of symptom sensitivity to hormone fluctuations or person-tailored and more effective treatment options for cyclicity of depressive symptoms. As of now, this study, along with others, highlights that the menstrual cycle needs to be considered when aiming at understanding depressive symptoms thoroughly.

## Electronic supplementary material

Below is the link to the electronic supplementary material.

Supplementary Material 1

Supplementary Material 2

## Data Availability

The datasets generated and/or analyzed during the current study are not publicly available due to privacy or ethical restrictions. All scripts for data preparation and analysis can be derived from the openly accessible R Script at https://osf.io/bfqc4/?view_only=be7992a4d6544eae8b8163383b058781.

## Footnotes

1 Item 3 (on sleep quality) was excluded from the sum-score since this item was only asked in the morning survey, after participants had slept.

## Abbreviations

LH: Luteinizing hormone
HPG axis: Hypothalamic-pituitary-gonadal axis
PMDD: Premenstrual dysphoric disorder
SCID-5-CV: Structural Clinical Interview for DSM-5 Disorders
PHQ: Patient Health Questionnaire
CI: 95% confidence interval
SD: Standard deviation
