# Supplementary material for: Menstrual cycle related depressive symptoms and their diurnal fluctuations – an ambulatory assessment study
Source: BMC Womens Health. 2024 Nov 18;24:611. doi: 10.1186/s12905-024-03438-9 (PMC11572536; doi:10.1186/s12905-024-03438-9)
Supplement: Supplementary file 2 — Supplementary Material 2 [file 12905_2024_3438_MOESM2_ESM.docx]

**Suplemental Material**

Appendix A: Detailed exclusion criteria (chronic disease and medication)

Appendix B: Modified PHQ-9 adapted for ambulatory assessment

Appendix C: Procedure for determining standardized cycle days

Appendix D: Flowchart of participant inclusion

Appendix E: Results of pairwise t-test to compare cyclicity of depressive symptoms

Appendix F: Results of t-test to compare cyclicity between depressed and non-depressed group

**Appendix A**

Excluded chronic diseases and medication.

Chronic disease or condition

Psoriasis

Lymph node impairment Endometriosis

Diabetes Type 1 / 2 Neurodermatitis

Asthma

AV Block

Polycystic Ovaries

Crohn's disease

Hashimoto

Coeliac disease

Lichen sclerosus

Glaucoma

Hay fever

Irritable bowel syndrome Grave's disease

Sinusitis

Anemia

Menier's disease

Pain syndrome

Blood clotting disorders Gastritis

Epilepsy

Alopecia areata

Medication

Glucocorticoids / hydrocortisone (continuous intake)

All psychotropic drugs

Anticonvulsants

Sedatives
Orraycea

**Appendix B**

Modified PHQ-9 adapted for ambulatory assessment

**German original:**

Wie sehr fühlen Sie sich **GERADE** durch die folgenden Beschwerden beeinträchtigt?

1) Wenig Interesse oder Freude an Ihren Tätigkeiten

2) Niedergeschlagenheit, Schwermut oder Hoffnungslosigkeit

3) Schwierigkeiten ein- oder durchzuschlafen oder vermehrter Schlaf

4) Müdigkeit oder Gefühl, keine Energie zu haben

5) Verminderter Appetit oder übermäßiges Bedürfnis zu essen

6) Schlechte Meinung von sich selbst; Gefühl, ein Versager zu sein oder die Familie enttäuscht zu haben

7) Schwierigkeiten, sich auf etwas zu konzentrieren, z.B. beim Zeitunglesen oder Fernsehen?

8) Sind Ihre Bewegungen oder Ihre Sprache so verlangsamt, dass es auch anderen auffallen würde? Oder sind Sie im Gegenteil „zappelig“ oder ruhelos und hatten dadurch einen stärkeren Bewegungsdrang als sonst?

9) Gedanken, dass Sie lieber tot wären oder sich Leid zufügen möchten

| gar nicht  0 | 1 | 2 | 3 | 4 | Sehr stark  5 |
| --- | --- | --- | --- | --- | --- |
|  |  |  |  |  |  |
| o | o | o | o | o | o |

**English translation:**

How much do you feel affected by the following symptoms RIGHT NOW?

1) Little interest or pleasure in your activities

2) Low spirits, melancholy or hopelessness

3) Difficulty falling asleep/staying asleep or increased sleep

4) Fatigue or feeling of having no energy

5) Decreased appetite or excessive need to eat

6) Poor opinion of self; feeling like a failure or having let family down

7) Difficulty concentrating on something, such as reading the newspaper or watching television

8) Are your movements or speech so slowed down that others would notice? Or, on the contrary, are you "fidgety" or restless and thus had a stronger urge to move than usual?

9) Thoughts that you would rather be dead or harm yourself

| Not at all  0 | 1 | 2 | 3 | 4 | Very strongly  5 |
| --- | --- | --- | --- | --- | --- |
|  |  |  |  |  |  |
| o | o | o | o | o | o |

**Appendix C**

Procedure for determining standardized cycle days

First, we assigned a forward count and a backward count variable to each assessment day of each participant as described by Schmalenberger et al. (2021): *forward count* is defined as the respective assessment date minus the previous cycle start date (start_cycle_1), therefore it typically ranges from 1 to 28. *Backward count* is defined as the assessment date minus the next cycle start plus 1 (start_cycle_2), therefore it typically ranges from -28 to -1. We further assigned an *ovulation count* variable to each day, with the day of positive ovulation test referred to as day zero, the prior days as -1, -2, -3 etc. and the subsequent days as 1,2,3 etc.

Second, we assigned one of the four cycle phases to respective assessment days: mid-follicular (days -7 to -3 before positive lh test or days 4 to 7 after menstrual onset (forward count)), periovulatory (days -2 to +1 around positive lh test or, days -15 to -12 based on backward count), mid-luteal (days -9 to -5 before menstrual onset (backwardcount) or premenstrual phase^[[1]](#footnote-1)^(three days before onset of menses (based on backwardcount)). For more background see the openly accessible R script, or the outstanding guideline paper by Schmalenberger et al. (2021).

Third, we selected 27 uniform days (c0 – c26) from each participant to be able to compare between and within effects of cyclicity.

For days c0 – c9, we selected the first ten days of the assessment cycle to represent the menstrual and mid-follicular hormone pattern (low estrogen and progesterone).

For days c10 – c16, we selected the seven days surrounding a positive LH test to represent the periovulatory hormone pattern (strong rise and fall of oestradiol and LH). If no ovulation test result was available, we estimated the periovulatory hormone pattern by using days -17 to -11 counting back from the onset of next menses.

For days c17 – c26, we used the last 10 days of the assessment cycle before onset of next menses, representing the mid-luteal and premenstrual hormone pattern (strong rise and fall of progesterone, slight rise and fall of estrogen). If a participant had a short cycle resulting in cycle days being assigned to multiple standardized days, the days reflecting the periovulatory phase (c10 – c16) were chosen over other phases. In summary, this resulted in a dataset of 27 cycle days per participant (newly assigned as c0 – c26), where the cycle days fell into comparable phases.

**Three examples for determining standardized cycle days**

28 day cycle with positive LH test on day 14/-15

| **Forward** | 1 | 2 | 3 | 4 | 5 | 6 | 7 | 8 | 9 | 10 | 11 | 12 | 13 | 14 | 15 | 16 | 17 | 18 | 19 | 20 | 21 | 22 | 23 | 24 | 25 | 26 | 27 | 28 |
| --- | --- | --- | --- | --- | --- | --- | --- | --- | --- | --- | --- | --- | --- | --- | --- | --- | --- | --- | --- | --- | --- | --- | --- | --- | --- | --- | --- | --- |
| **Backward** | -28 | -27 | -26 | -25 | -24 | -23 | -22 | -21 | -20 | -19 | -18 | -17 | -16 | -15 | -14 | -13 | -12 | -11 | -10 | -9 | -8 | -7 | -6 | -5 | -4 | -3 | -2 | -1 |
| **Ovulation** |  |  |  |  |  |  |  |  |  |  | -3 | -2 | -1 | 0 | 1 | 2 | 3 |  |  |  |  |  |  |  |  |  |  |  |
| **Cycle day** | c0 | C1 | C2 | C3 | C4 | C5 | C6 | C7 | C8 | C9 | C10 | C11 | C12 | C13 | C14 | C15 | C16 |  | C17 | C18 | C19 | C20 | C21 | C22 | C23 | C24 | C25 | C26 |

32 day cycle with positive LH test on day 18/-15

| **Forward** | 1 | 2 | 3 | 4 | 5 | 6 | 7 | 8 | 9 | 10 | 11 | 12 | 13 | 14 | 15 | 16 | 17 | 18 | 19 | 20 | 21 | 22 | 23 | 24 | 25 | 26 | 27 | 28 | 29 | 30 | 31 | 32 |
| --- | --- | --- | --- | --- | --- | --- | --- | --- | --- | --- | --- | --- | --- | --- | --- | --- | --- | --- | --- | --- | --- | --- | --- | --- | --- | --- | --- | --- | --- | --- | --- | --- |
| **Backward** | -32 | -31 | -30 | -29 | -28 | -27 | -26 | -25 | -24 | -23 | -22 | -21 | -20 | -19 | -18 | -17 | -16 | -15 | -14 | -13 | -12 | -11 | -10 | -9 | -8 | -7 | -6 | -5 | -4 | -3 | -2 | -1 |
| **Ovulation** |  |  |  |  |  |  |  |  |  |  |  |  |  |  | -3 | -2 | -1 | 0 | 1 | 2 | 3 |  |  |  |  |  |  |  |  |  |  |  |
| **Cycle day** | c0 | C1 | C2 | C3 | C4 | C5 | C6 | C7 | C8 | C9 |  |  |  |  | C10 | C11 | C12 | C13 | C14 | C15 | C16 |  | C17 | C18 | C19 | C20 | C21 | C22 | C23 | C24 | C25 | C26 |

25 day cycle with positive LH test on day 12/-14

| **Forward** | 1 | 2 | 3 | 4 | 5 | 6 | 7 | 8 | 9 | 10 | 11 | 12 | 13 | 14 | 15 | 16 | 17 | 18 | 19 | 20 | 21 | 22 | 23 | 24 | 25 |
| --- | --- | --- | --- | --- | --- | --- | --- | --- | --- | --- | --- | --- | --- | --- | --- | --- | --- | --- | --- | --- | --- | --- | --- | --- | --- |
| **Backward** | -25 | -24 | -23 | -22 | -21 | -20 | -19 | -18 | -17 | -16 | -15 | -14 | -13 | -12 | -11 | -10 | -9 | -8 | -7 | -6 | -5 | -4 | -3 | -2 | -1 |
| **Ovulation** |  |  |  |  |  |  |  |  | -3 | -2 | -1 | 0 | 1 | 2 | 3 |  |  |  |  |  |  |  |  |  |  |
| **Cycle day** | c0 | C1 | C2 | C3 | C4 | C5 | C6 | C7 | C10 | C11 | C12 | C13 | C14 | C15 | C16 | C17 | C18 | C19 | C20 | C21 | C22 | C23 | C24 | C25 | C26 |

🡪 this person would have no values for days C8 and C9 because of a shorter follicular phase

**Appendix D**

Flowchart of participant inclusion


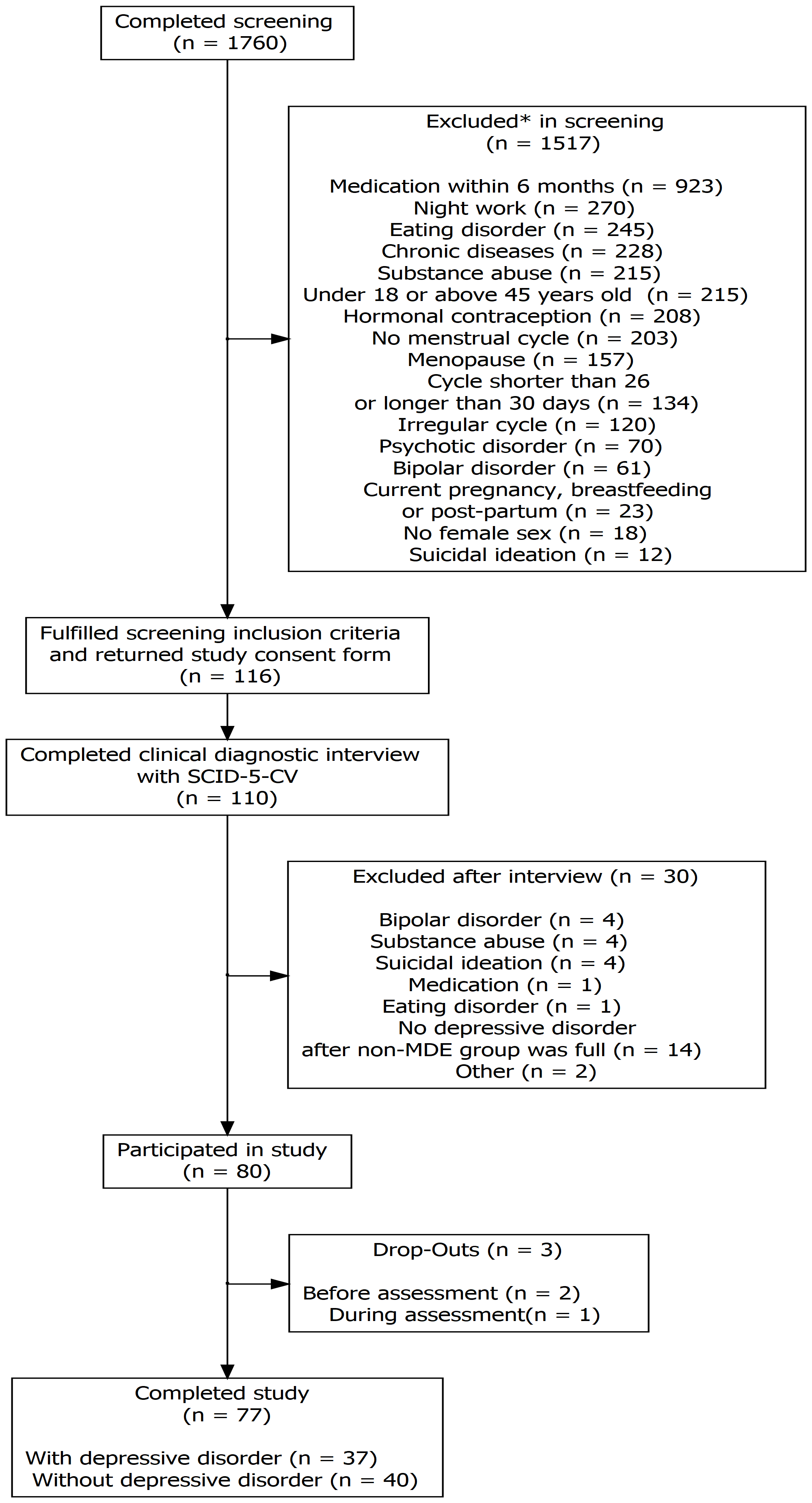


**Appendix E**

Results of pairwise t-test to compare cyclicity of depressive symptoms

| Symptom 1 | Symptom 2 | Pairwise comparison | Adjusted p-value | Significance adjustet p-value | Mean cc symptom 1 | Mean cc symptom 2 |
| --- | --- | --- | --- | --- | --- | --- |
| Changed appetite | Concentration problems | t(76)=4.294 , *p* < .001 | >0.01 | ** | 0.01 | 0.08 |
| Changed appetite | Depressed mood | t(76)=-0.033 , *p* = .974 | 1.00 | ns | 0.01 | 0.32 |
| Changed appetite | Diminished interest | t(76)=3.796 , *p* < .001 | 0.01 | ** | 0.01 | 0.08 |
| Changed appetite | Low energy | t(76)=0.684 , *p* = .496 | 1.00 | ns | 0.01 | 0.19 |
| Changed appetite | Reduced movement/Restlessness | t(76)=5.437 , *p* < .001 | >0.01 | **** | 0.01 | 0.13 |
| Changed appetite | Suicidal ideation | t(76)=10.093 , *p* < .001 | >0.01 | **** | 0.01 | 0.06 |
| Changed appetite | Feeling worthless | t(76)=4.751 , *p* < .001 | >0.01 | *** | 0.01 | 0.12 |
| Concentration problems | Depressed mood | t(76)=-4.385 , *p* < .001 | >0.01 | ** | 0.08 | 0.32 |
| Concentration problems | Diminished interest | t(76)=-0.362 , *p* = .718 | 1.00 | ns | 0.08 | 0.08 |
| Concentration problems | Low energy | t(76)=-4.135 , *p* < .001 | >0.01 | ** | 0.08 | 0.19 |
| Concentration problems | Reduced movement/Restlessness | t(76)=1.886 , *p* = .063 | 1.00 | ns | 0.08 | 0.13 |
| Concentration problems | Suicidal ideation | t(76)=8.029 , *p* < .001 | >0.01 | **** | 0.08 | 0.06 |
| Concentration problems | Feeling worthless | t(76)=1.369 , *p* = .175 | 1.00 | ns | 0.08 | 0.12 |
| Depressed mood | Diminished interest | t(76)=4.965 , *p* < .001 | >0.01 | *** | 0.32 | 0.08 |
| Depressed mood | Low energy | t(76)=0.843 , *p* = .402 | 1.00 | ns | 0.32 | 0.19 |
| Depressed mood | Reduced movement/Restlessness | t(76)=5.921 , *p* < .001 | >0.01 | **** | 0.32 | 0.13 |
| Depressed mood | Suicidal ideation | t(76)=10.353 , *p* < .001 | >0.01 | **** | 0.32 | 0.06 |
| Depressed mood | Feeling worthless | t(76)=6.431 , *p* < .001 | >0.01 | **** | 0.32 | 0.12 |
| Diminished interest | Low energy | t(76)=-5.216 , *p* < .001 | >0.01 | **** | 0.08 | 0.19 |
| Diminished interest | Reduced movement/Restlessness | t(76)=2.506 , *p* = .014 | 0.40 | ns | 0.08 | 0.13 |
| Diminished interest | Suicidal ideation | t(76)=13.632 , *p* < .001 | >0.01 | **** | 0.08 | 0.06 |
| Diminished interest | Feeling worthless | t(76)=2.178 , *p* = 0.032 | 0.91 | ns | 0.08 | 0.12 |
| Low energy | Reduced movement/Restlessness | t(76)=6.438 , *p* < .001 | >0.01 | **** | 0.19 | 0.13 |
| Low energy | Suicidal ideation | t(76)=14.991 , *p* < .001 | >0.01 | **** | 0.19 | 0.06 |
| Low energy | Feeling worthless | t(76)=6.799 , *p* < .001 | >0.01 | **** | 0.19 | 0.12 |
| Reduced movement/Restlessness | Suicidal ideation | t(76)=5.563 , *p* < .001 | >0.01 | **** | 0.13 | 0.06 |
| Reduced movement/Restlessness | Feeling worthless | t(76)=-0.896 , *p* = 0.373 | 1.00 | ns | 0.13 | 0.12 |
| Suicidal ideation | Feeling worthless | t(76)=-9.515 , *p* < .001 | >0.01 | **** | 0.06 | 0.12 |
| Note: CC = cosine coefficient | | | | | | |

**Tile plot of visualizing effect sizes of pairwise comparisons**


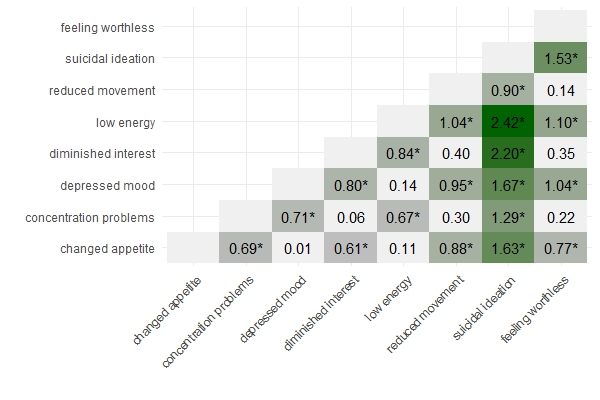


Note: *significant comparisons marked with “*”, darker green displays larger effect sizes

**Appendix F**

Comparison of cosine coefficients between depressed/non-depressed

| Symptom | p-value | Statistically significant difference | Effect size (d) | Mean (SD) depressed | Mean (SD) not depressed |
| --- | --- | --- | --- | --- | --- |
| Diminished interest | 0.04 | TRUE | 0.47 | 0.148 (0.067) | 0.114 (0.076) |
| Depressed mood | 0.45 | FALSE | -0.17 | 0.199 (0.167) | 0.229 (0.174) |
| Low energy | 0.13 | FALSE | -0.34 | 0.18 (0.094) | 0.217 (0.121) |
| Changed appetite | 0.54 | FALSE | -0.14 | 0.201 (0.157) | 0.225 (0.189) |
| Feeling worthless | 0.63 | FALSE | -0.11 | 0.104 (0.077) | 0.114 (0.101) |
| Concentration problems | 0.67 | FALSE | -0.10 | 0.122 (0.1) | 0.133 (0.146) |
| Reduced movement/Restlessness | 0.09 | FALSE | -0.39 | 0.073 (0.104) | 0.122 (0.147) |
| Suicidal ideation | 0.00 | TRUE | -0.65 | 0.008 (0.009) | 0.023 (0.03) |
| Sum score | 0.56 | FALSE | -0.13 | 0.873 (0.61) | 0.96 (0.709) |
| Note: CC = cosine coefficient. Effect size (d) = Cohen’s d. Table shows comparison of absolute cosine coefficients and if normal distribution and variance homogeneity for t-test was fulfilled. Sumscore is calculated of all PHQ-items except item 3 (sleep problems). Item 3 was not asked in the afternoon survey. | | | | | |

1. As we assessed only one cycle, starting with the first days of menses, we did not assess the perimenstrual phase (which measures the 5 days sourrounding each menstrual start, but the premenstrual phase at the end of the assessment) [↑](#footnote-ref-1)
